# “I’d Do Things to You That You Can’t Even Imagine, but You’d Love Them …”: Situating #MeToo Among Spanish Youth

**DOI:** 10.3390/bs15121607

**Published:** 2025-11-21

**Authors:** Tomás Cámara-Pastor, Javier Ortuño-Sierra, Andrea Gutiérrez-García

**Affiliations:** Department of Educational Sciences, University of La Rioja, C/Luis de Ulloa, 2, 26004 Logroño, La Rioja, Spainandrea.gutierrezg@unirioja.es (A.G.-G.)

**Keywords:** sexual violence, sexual harassment, gender-based violence, LGBTIQ+, higher education, Spain

## Abstract

Silence has historically been the greatest ally of sexual violence (SV); the #MeToo movement disrupted this silence on a global scale through an unprecedented wave of collective denunciation. Sexualizing behaviors—including intrusive gazes, inappropriate cues, and non-consensual contact—have become pervasive, often exacerbated by the reach of digital technologies and social media. Accordingly, the primary objective of this study was to provide empirical data on SV victimization among young people in Spain. A total of 1102 anonymous college students (aged 17–30; *M* = 22.22, *SD* = 2.09) completed a survey administered via the Qualtrics platform. Data were subsequently analyzed using R software. This study (1) reports retrospective and recent prevalence rates of (non-)partner SV and sexual harassment; (2) highlights the most common forms of perpetration; (3) determines the age at which the first incident occurs; (4) identifies the most frequent perpetrators by SV type and victim characteristics; and (5) examines sociodemographic correlates by gender, age, and sexual orientation using a cross-sectional analysis approach. Finally, acknowledging methodological limitations, the psychological, social, and political implications of the findings are discussed, underscoring the urgency of recognizing key victimological profiles through an intersectional lens that incorporates sexual and gender diversity.

## 1. Introduction

Sexual violence constitutes one of the most severe violations of human rights and remains a critical global public health concern. The statistics are striking: it is estimated that one in four women have experienced sexual violence by an intimate partner over the course of her life (World Health Organization, 2021). Among girls and adolescents, prevalence rates range from 18% to 24%, with even more alarming figures reported in regions such as South America and sub-Saharan Africa (Cagney et al., 2025). These data highlight the urgent need to address patterns of violence that often emerge early in life and have lasting repercussions on psychological well-being.

Late adolescence and emerging adulthood represent periods of heightened vulnerability, marked by profound psychosocial transitions and the shift toward greater autonomy. These stages are often accompanied by increased exposure to intimate interactions that may evolve into patterns of control or victim–perpetrator dynamics (Arnett, 2000; Johnson et al., 2015). A more nuanced examination of these developmental phases is essential, taking into account factors such as identity formation, the restructuring of support networks, and the internalization of relational norms.

Specifically, Partner Sexual Violence (PSV) refers to any non-consensual sexual act perpetrated by a current or former intimate partner. Non-Partner Sexual Violence (NPSV) includes similar acts committed by individuals with whom the victim does not have an intimate relationship. Sexual Harassment (SH) encompasses unwanted sexual behaviors—verbal, non-verbal, or physical—that create a hostile or intimidating environment (Abrahams et al., 2014; Bagwell-Gray et al., 2015; Barker et al., 2019). Recent meta-analyses reveal that up to 41% of adult women report having experienced PSV at some point in their lives, while the combined prevalence of PSV and NPSV exceeds 20% among adolescents (Cagney et al., 2025; Pereda et al., 2016). Furthermore, European studies indicate that between 40% and 60% of adolescent girls have encountered sexual harassment either online or in person, underscoring the urgent need for preventive educational policies rooted in school and community settings (e.g., Melendez-Torres et al., 2024; Sakellari et al., 2022).

The growing availability of psychometrically sound instruments, such as the *Perceived Severity of Adolescent Dating Violence* scale (PS-ADV; Arrojo et al., 2024), has enhanced the ability to assess the severity and emotional impact of violent relational experiences with greater precision. At the same time, emerging phenomena like digital sexualization, particularly sexting, have been linked to an increased risk of experiencing sexual violence among youth (Bonsaksen et al., 2024; Kaufman et al., 2021; Ybarra et al., 2016). In this context, the COVID-19 pandemic exposed the syndemic nature of sexual violence by amplifying risk factors such as social isolation, declining mental health, and substance use, especially among LGBTIQ+ youth (Costa et al., 2024; Nacht et al., 2025). In Spain, recent data from the Government Delegation for Gender-Based Violence (2023) indicate that substances were involved in 40% of reported sexual assaults, chemical submission (refers to the administration of psychoactive substances to a victim without their knowledge or consent, with the purpose of facilitating sexual assault (Government Delegation for Gender-Based Violence, 2023)) occurred in 13% of cases, and nearly one-third of the perpetrators were minors or acted in groups, trends that demand an urgent recalibration of prevention policies.

Recent epidemiological research estimates that, as of 2023, the global prevalence of sexual violence against minors reached 18.9% among girls and 14.8% among boys (Cagney et al., 2025). These figures underscore a deeply concerning reality, further compounded by the psychological consequences frequently associated with early victimization, including anxiety, post-traumatic stress disorder, mood disorders, and other long-lasting mental health effects (Sakellari et al., 2022; Ybarra et al., 2016). From a theoretical standpoint, Bronfenbrenner’s (1979) ecological model offers a valuable framework for understanding sexual violence across multiple levels—individual, relational, community, and societal—supporting the design of multilayered interventions tailored to youth contexts. Simultaneously, Arnett’s (2000) theory of emerging adulthood elucidates how this developmental period entails increased engagement in intimate relationships, thereby amplifying the risk of experiencing sexual victimization.

Recent studies highlight that late adolescence and emerging adulthood constitute periods of heightened vulnerability, driven by profound psychosocial changes (Arnett, 2000; Johnson et al., 2015). These developmental stages are characterized by increasing autonomy in interpersonal relationships and greater exposure to sexual violence dynamics, particularly among university students and individuals belonging to sexual minority groups (Garcia-Moreno et al., 2006; Walters et al., 2013). Social movements such as #MeToo have brought to light entrenched structural and cultural barriers that hinder both the recognition and reporting of sexual harassment (Hegarty & Tarzia, 2019; Kaufman et al., 2021). Despite this progress, significant methodological challenges remain, including the absence of standardized definitions, the influence of social normalization biases, and fear of retaliation, all of which contribute to the underestimation of the true prevalence of sexual violence (Leskinen et al., 2011; Ybarra et al., 2016).

Bronfenbrenner’s ecological model conceptualizes human development as the result of interactions between individuals and multiple environmental systems (micro-, meso-, exo-, and macrosystem) (Bronfenbrenner, 1979). Applied to sexual violence, the microsystem includes the immediate relational contexts in which victimization occurs—such as romantic partnerships, peer groups, and university settings—where factors like the perpetrator’s endorsement of hostile masculinity, alcohol and substance use, and limited knowledge or skills regarding consent may increase the likelihood of abuse. At the mesosystem level, the interplay between family, peers, and educational institutions can either buffer or intensify risk; for example, inconsistent messages regarding sexuality and gender equality, weak bystander cultures within peer networks, or institutional minimization of complaints may normalize coercive behaviors. The exosystem comprises distal structures that shape opportunities and constraints for both victims and perpetrators, including the proliferation of sexualized digital content, precarious working conditions, and variable institutional responses to reports of violence. Finally, macrosystem factors—such as gendered socialization, heteronormative cultural scripts, and broader rape-supportive norms—provide the structural backdrop that legitimizes or challenges sexual violence. Ecological approaches to sexual violence emphasize this multilevel interplay of determinants and underscore the need to situate individual experiences of (non-)partner sexual violence and harassment within nested social systems (Moylan & Javorka, 2020; Stockman et al., 2023). Similarly, Arnett’s (2000) theory of emerging adulthood describes the developmental stage between adolescence and full adulthood (approximately 18–29 years), characterized by identity exploration, instability, and increased autonomy, conditions that may heighten vulnerability to sexual victimization (Arnett, 2000). In this life stage, young people typically experiment with intimate and sexual relationships, transition between educational and occupational settings, and expand their engagement with digital communication and social media, all of which increase both opportunities for consensual intimacy and exposure to coercive or unwanted sexual encounters. This combined ecological and developmental lens informed the selection of the age range (17–30 years), the distinction between partner and non-partner sexual violence, the emphasis on sexual harassment behaviors in everyday social and institutional settings, and the examination of gender, age, and sexual orientation as key sociodemographic correlates in the present study.

Within this context, the present study primarily aimed to provide updated empirical data on sexual violence victimization among young people in Spain. The specific objectives were as follows: (1) to estimate both lifetime and recent prevalence rates of PSV, NPSV, and SH; (2) to identify the most common forms of perpetration; (3) to determine the age at which the first incident occurs; (4) to profile perpetrators; and (5) to examine sociodemographic correlates—including gender, age, and sexual orientation—using a cross-sectional analytical approach.

## 2. Materials and Methods

### 2.1. Participants and Procedure

This study employed a cross-sectional design with a total sample of 1102 participants aged 17 to 30 years (*M* = 22.22, *SD* = 2.09). The final sample comprised 1102 participants: 66.6% women (*n* = 754) and 33.4% men (*n* = 345). Regarding sexual orientation, 81.4% identified as heterosexual (*n* = 895) and 18.6% as LGBTIQ+ (*n* = 204). Age distribution was as follows: 17–19 years (42.9%, *n* = 471), 20–24 years (53.2%, *n* = 585), and 25–30 years (3.9%, *n* = 43). Recruitment was conducted through undergraduate programs at several Spanish universities. Although predominantly composed of university students, the sample was conceptualized as representative of the developmental stages of late adolescence and emerging adulthood. Eligibility criteria included residence in Spain and age between 17 and 30 years. Participation was fully voluntary, anonymous, and confidential. To ensure data integrity and anonymity, each participant received a unique alphanumeric identifier.

Ethical approval was obtained from the University of La Rioja Research Ethics Committee (Ref. CEUR-2023-045; 12 July 2023). Informed consent was secured from all participants aged 18 and above. For those aged 17, digital consent from parents or legal guardians and assent from adolescents were obtained prior to data collection. All data were stored on encrypted servers accessible solely by the research team, in strict compliance with GDPR regulations.

The survey was conducted online via institutional platforms and social media channels associated with participating universities. Faculty members were invited to distribute the survey link during class time or through internal mailing lists. The self-administered questionnaire was hosted on the Qualtrics^®^ platform and required approximately 15 to 20 min. Prior to participation, respondents were informed of the voluntary of the study, confidentiality assurances, and their right to withdraw at any time without repercussion. Data collection occurred from March to June 2024.

### 2.2. Instruments

#### 2.2.1. (Non-)Partner Sexual Violence

PSV and NPSV were assessed using 7 items adapted from the Spanish National Survey on Violence Against Women. Examples include: ‘Has a partner ever forced you to engage in sexual activity against your will?’ (PSV) and ‘Has someone outside a romantic relationship ever touched you in a sexual way without your consent?’ (NPSV) (Government Delegation for Gender-Based Violence, 2019). Participants indicated whether they had experienced specific forms of sexual violence (e.g., unwanted sexual contact, coerced sexual acts) at any time during their lifetime and within the previous 12 months. Response options included “no,” “yes,” and “prefer not to say”. For some analyses, responses were dichotomized (0 = no; 1 = yes), excluding cases where participants chose the “prefer not to say” option. Although some behaviors (e.g., lewd gazes, offensive comments) may resemble sexual harassment, their classification under PSV or NPSV was determined by the relational context (partner vs. non-partner), in line with operational definitions provided above.

#### 2.2.2. Sexual Harassment

SH was assessed using 10 items, adapted from the previously cited Spanish survey, representing various unwanted sexual behaviors such as persistent staring, unsolicited explicit messages, offensive jokes, and unwanted physical contact. Responses were coded as 0 (no), 1 (yes), or 2 (prefer not to say). For analytical purposes, participants were classified as having experienced SH if they endorsed at least one behavior with a positive response (1 = yes), while responses of “prefer not to say” were preserved to honor participant autonomy. The items were grouped into specific categories to represent various dimensions of sexual harassment, including intimidation (unwanted staring), exhibitionism (inappropriate comments or gestures), exposure to unsolicited sexual material (inappropriate content), unwelcome advances (unwanted sexual propositions), inappropriate contact (unwelcome physical proximity or touch), and workplace harassment, which refers to sexual misconduct occurring in professional settings.

#### 2.2.3. Timing and Perpetrators

Participants reported the timing of these incidents using categories adapted from the Spanish survey, indicating whether the events occurred before age 15 (coded as 1), after age 15 (coded as 2), during both periods (coded as 3), or never (coded as 0). Additionally, they specified whether the incidents had taken place within the previous 12 months (no/yes). Perpetrator identity was assessed via 13 predefined categories (e.g., friend, classmate, stranger, romantic partner), with participants also identifying the perpetrator’s gender as male, female, or not applicable.

#### 2.2.4. Sociodemographic Factors

Sociodemographic data were collected on gender (man, woman), age (17–19, 20–24, and 25–30 years), and sexual orientation (heterosexual, LGBTIQ+).

### 2.3. Statistical Analyses

Descriptive analyses were conducted to estimate the prevalence of PSV, NPSV, and SH across the entire sample and stratified by key sociodemographic variables, including gender (man/woman), age group (17–19, 20–24, and 25–30 years), and sexual orientation (heterosexual/LGBTIQ+). Bivariate relationships between sexual victimization and these sociodemographic factors were assessed using chi-square (*χ*^2^) tests, with significance established at *p* < 0.05. Furthermore, a detailed item-level evaluation examined the distribution of specific sexual violence behaviors within the three domains (PSV, NPSV, and SH). For each behavior, frequencies (*n*, %) were computed and disaggregated by gender, age group, and sexual orientation. Chi-square tests of independence were again employed to determine statistically significant differences in prevalence according to these categories, applying corrections when small expected counts or low frequencies warranted adjustments. The temporal dimension of victimization was also explored, differentiating incidents occurring before age 15, after age 15, during both periods, or never, along with occurrences reported within the previous 12 months.

Multivariate binary logistic regression models were utilized to identify independent predictors of sexual victimization, treating victimization status as a binary outcome (0 = no, 1 = yes). Predictors included gender (0 = man, 1 = woman), age category (0 = 17–19, 1 = 20–24, 2 = 25–30 years), and sexual orientation (0 = heterosexual, 1 = LGBTIQ+). Participants who selected “prefer not to say” were excluded from these analyses. Adjusted odds ratios (OR) and 95% confidence intervals (CI) were calculated for each variable, and model adequacy was assessed through maximum likelihood estimation. Separate models were fit for each type of sexual violence: PSV analyses incorporated only individuals reporting a current or past romantic partner; NPSV analyses were limited to participants without such partners; SH analyses included the entire sample without exclusions.

All statistical procedures were carried out using R software, version 4.4.2 (R Core Team, 2023), with statistical significance defined as *p* < 0.05. Post hoc power calculations confirmed adequate power (≥80%) to detect medium effect sizes (OR ≥ 1.5) based on the sample size and observed prevalence of SH.

## 3. Results

### 3.1. Let’s Talk About (Non-)Partner Sexual Violence

Across the full sample of 1102 individuals aged 17–30, 11.9% reported having been subjected to PSV at some point in their lives, while 18.9% disclosed incidents of NPSV (Table 1). Regarding the timing of these occurrences, only 0.6% of participants reported PSV before the age of 15, compared to 8.8% after the age of 15; an additional 2.4% were exposed to PSV during both developmental periods. Similarly, 1.5% experienced NPSV prior to age 15, 14.1% after that age of 15, and 3.1% during both stages. Within the past 12 months, 5.2% acknowledged recent episodes of PSV, while 7.9% reported NPSV.

Bivariate analyses revealed that women were significantly more likely than men to report experiences of PSV (15.4% vs. 4.3%; *χ*^2^, *p* < 0.001), and that LGBTIQ+ participants exhibited significantly higher rates of NPSV compared to heterosexual individuals (28.4% vs. 16.8%; *p* < 0.001). Although the 20–24 age group showed the highest PSV prevalence, this difference did not reach statistical significance (*p* = 0.073).

Table 2 details specific forms of PSV assessed alongside their sociodemographic correlates, including gender, age and sexual orientation. PSV demonstrated notably high prevalence, with the most endorsed behavior being “persistent or lewd gazes” (79.6%), followed by “unwanted physical contact” (73.5%) and “offensive sexual jokes or comments” (73.5%). Statistically significant differences by gender emerged in six of the ten items, consistently indicating higher rates among female participants. No significant variations were observed in relation to sexual orientation or age group. Additionally, over 55% of incidents were reported to have occurred within the past 12 months.

As presented in Table 3, regarding NPSV, the most frequently reported behaviors included “persistent or lewd gazes” (63.1%), followed by “offensive sexual jokes/comments” (46.4%) and “unwanted physical contact” (42.7%). Across most items, women exhibited significantly higher prevalence compared to men. Significant differences by sexual orientation were identified in six out of ten items, with LGBTIQ+ individuals showing increased vulnerability. Concerning age, only one item—“inappropriate sexual messages”—displayed a statistically significant difference. Most incidents occurred recently (within the past 12 months), underscoring the persistent and ongoing nature of this issue.

Logistic regression analyses adjusting for sex, age group, and sexual orientation (see Table 4) revealed that women had significantly higher odds of experiencing PSV compared to men (OR = 2.58; 95% CI = 1.50–4.75; *p* = 0.001). Participants aged 25–30 were also more likely to report PSV than those in the 17–19 age group (OR = 3.33; 95% CI = 1.34–7.58; *p* = 0.006), suggesting an age-related increase in risk. In the case of NPSV, female participants again demonstrated a markedly elevated likelihood of victimization (OR = 6.04; 95% CI = 4.56–8.07; *p* < 0.001), whereas age did not emerge as a significant predictor. Notably, sexual orientation did not significantly contribute to the prediction of either form of sexual violence in the hypothetical models.

Furthermore, analysis of perpetrator characteristics indicated that, in NPSV cases, the most frequently identified perpetrators were individuals “known by sight”, followed by strangers and friends. Overall, 83.6% of NPSV perpetrators were male. In PSV incidents, male assailants predominated even more markedly, accounting for 90.1% of cases (Table 5).

### 3.2. Let’s Now Talk About Sexual Harassment

SH emerged as the most prevalent form of sexual violence, with 73.2% of participants (*n* = 807) reporting at least one incident. As detailed in Table 6, lifetime prevalence rates of SH differed significantly across gender, age group, and sexual orientation. Women were markedly more likely than men to report experiences of harassment (77.9% vs. 54.3%), while men were more likely to report no such experiences (45.7% vs. 8.3%) or to choose not to disclose this information (5.8% vs. 6.7%). Regarding age, the highest prevalence was found among individuals aged 17–19 (71.7%) and 20–24 (72.5%), with a slight decrease observed in the 25–30 age group (66%). In terms of sexual orientation, participants identifying as LGBTIQ+ reported substantially higher rates of SH compared to their heterosexual counterparts (49.0% vs. 30.2%), underscoring a notable disparity in victimization across sexual orientation categories. Across all subgroups, a small percentage of participants opted not to respond (up to 8.4%), which may reflect persisting stigma, discomfort, or perceived risk associated with disclosing experiences of SH.

When focusing on the past 12 months, SH was reported by 57.5% of women, compared to 23.5% of men. Additionally, 40.0% of women stated they had not experienced harassment during the previous year, while 18.0% of men indicated the same. Strikingly, only 2.5% of women reported never having experienced SH, in contrast to 58.5% of men who stated they had never been subjected to such behavior.

A further analysis of SH timing by gender revealed distinct patterns. A greater proportion of women than men reported incidents occurring after the age of 15 (57.7% vs. 42.8%), as well as experiences spanning both childhood and adolescence/adulthood (30.7% vs. 15.6%). Reports of harassment occurring before age 15 were slightly more common among women (5.6%) than among men (4.2%). Conversely, men were significantly more likely than women to report never having been exposed to SH (37.4% vs. 6%).

Table 7 outlines the specific forms of SH reported. The most frequently endorsed behaviors included “unwanted touching of intimate body parts” (25.1%), “attempts to coerce sexual intercourse” (12.8%), and “being forced to touch another’s intimate body parts” (12.0%). Statistically significant gender differences were identified in nearly all forms of harassment, with women consistently reporting higher prevalence rates. Likewise, individuals identifying as LGBTIQ+ exhibited significantly greater vulnerability across all items compared to their heterosexual peers. Age-related disparities were observed in four of the seven behaviors assessed, with the highest prevalence again concentrated among participants aged 17–24. Notably, more than 60% of incidents were reported as having occurred within the past year.

Multivariate logistic regression models (Table 8) corroborated the observed trends in SH exposure. Compared to men, women exhibited significantly higher odds of reporting SH, with nearly a threefold increase in likelihood (OR = 2.78; 95% CI = 2.05–3.81; *p* < 0.001). Age also emerged as a relevant factor: participants aged 20–24 were significantly more likely than those aged 17–19 to report harassment experiences (OR = 1.65; 95% CI = 1.26–2.16; *p* < 0.001). Although individuals in the 25–30 age group also showed an increased probability, the association did not reach statistical significance. Additionally, identifying as part of the LGBTIQ+ community was associated with a twofold increase in the likelihood of reporting SH (OR = 2.00; 95% CI = 1.45–2.75; *p* < 0.001), underscoring the heightened vulnerability of sexual and gender minorities to these forms of victimization.

## 4. Discussion

To facilitate interpretation, the different forms of sexual violence assessed in this study can be grouped into three main categories: (a) verbal and non-verbal harassment (e.g., lewd gazes, offensive comments), (b) unwanted physical contact (e.g., touching intimate body parts), and (c) coercive or forced sexual acts (e.g., attempts to force intercourse). This classification underscores the continuum of behaviors ranging from subtle harassment to severe sexual assault, highlighting the need for multidimensional prevention strategies.

The present study primarily aimed to provide updated empirical data on sexual violence victimization among young people in Spain. Next, the findings associated with each specific objective are discussed in accordance with the existing scientific literature:

The first specific objective was to estimate both lifetime and recent prevalence rates of PSV, NPSV, and SH. The findings reveal a substantial burden of victimization, with SH emerging as the most pervasive form, followed by NPSV and PSV. A significant proportion of these incidents occurred within the past year, suggesting not only the persistence but also the immediacy of these forms of violence during a developmental period characterized by identity exploration, relational reconfiguration, and increased psychosocial vulnerability. When situated within international epidemiological literature, these findings are consistent with estimates from large-scale systematic reviews and meta-analyses. For instance, global data indicate that up to 41% of adult women report experiences of PSV during their lifetime, and the combined prevalence of PSV and NPSV in adolescents typically exceeds 20% (Cagney et al., 2025; Pereda et al., 2016). Our observed rates fall within this expected range, though notably elevated in the case of SH, which affected over 70% of participants—surpassing figures reported in recent European studies that estimate prevalence between 40% and 60% among adolescent girls (Piolanti et al., 2025; Sakellari et al., 2022). These patterns suggest that SH not only persists but may escalate in frequency during emerging adulthood.

The second specific objective of the present study was to identify the most common forms of perpetration in cases of PSV and NPSV. Findings indicate that the most prevalent behaviors include “persistent or lewd gazes,” “unwanted physical contact,” and “offensive sexual jokes or comments” across both PSV and NPSV contexts. These results align with recent literature emphasizing the widespread presence of sexual harassment and microaggressions as frequent and early manifestations of sexual violence in young populations (Fitzgerald & Cortina, 2018; Klein & Martin, 2021). Systematic reviews highlight that although such behaviors are often socially normalized, they constitute risk factors for escalation to more severe forms of sexual assault and have significant psychological consequences, including anxiety, depression, and complex trauma (Dokkedahl et al., 2019; Mellen et al., 2024). The high prevalence of “lewd gazes” and “offensive comments” underscores the need to extend preventive efforts beyond overt physical aggression to also address verbal and gestural behaviors that perpetuate climates of insecurity and social control over victims (Aji et al., 2024). From social and clinical psychology perspectives, these perpetration forms are grounded in power dynamics and gender norms that legitimize objectification and domination, necessitating interventions including educational and workplace-based awareness and re-education programs promoting equality, respect, and empathy (Stewart et al., 2021; Villardón-Gallego et al., 2023). In summary, the perpetration pattern identified in this study highlights the importance of developing multidimensional preventive strategies that consider the subtle yet harmful nature of common forms of sexual violence and their impact on mental health and social well-being among youth.

The third specific objective of the present study was to determine the age at which the first incident of sexual violence occurs. Findings suggest that while a minority of participants reported victimization during childhood, the majority of incidents—across all forms of sexual violence—were first experienced after the age of 15, with a considerable portion occurring during adolescence and emerging adulthood. These results align with developmental research emphasizing the transitional vulnerability of this life stage, characterized by increased autonomy, relational experimentation, and identity formation (Arnett, 2000; Johnson et al., 2015). A growing body of evidence, including longitudinal and meta-analytic reviews, underscores the heightened risk of initial sexual victimization during adolescence and young adulthood, periods marked by both biological maturation and intensified peer interactions (Grummitt et al., 2024; Stockman et al., 2023). For example, a recent meta-analysis by Assink et al. (2019) indicates that the onset of sexual victimization peaks between ages 15 and 25, a developmental window consistently identified as a critical period for both prevention and early intervention. These developmental trajectories support the implementation of age-tailored preventive frameworks in secondary and postsecondary education settings. Moreover, evidence from trauma psychology highlights that early exposure to sexual violence—even in adolescence—can significantly disrupt cognitive-emotional development, increase vulnerability to revictimization, and contribute to long-term psychopathology (Cook et al., 2005; Trickett et al., 2011). Therefore, the present findings not only confirm the temporal concentration of initial incidents during late adolescence but also emphasize the psychological imperative to intervene during this developmental window through empirically grounded, trauma-informed strategies.

The fourth specific objective of the present study was to profile the perpetrators of PSV and NPSV. Findings revealed that perpetrators in NPSV cases were predominantly individuals “known by sight”—such as acquaintances—followed by strangers and friends, with males accounting for the vast majority of offenders (83.6% in NPSV, 90.1% in PSV). This distribution aligns with contemporary epidemiological research highlighting that sexual violence is overwhelmingly perpetrated by males within the victim’s social or relational milieu rather than by strangers (O’Connor et al., 2024; Thomas et al., 2022). A recent systematic review examining characteristics of sexual violence perpetrators underscored the importance of relational proximity, demonstrating that familiarity facilitates access and opportunity for abuse, which has implications for risk assessment and prevention strategies (Lacambre & Bodkin, 2023). Psychologically, these findings reinforce the need for preventive interventions that extend beyond stranger-danger paradigms to include relational and social contexts where boundaries are often blurred. Thus, empirically informed programs focusing on enhancing interpersonal skills, consent education, and bystander intervention in peer and community networks are critical for disrupting cycles of victimization and perpetration (e.g., Blackburn et al., 2024; Orr et al., 2022; Park & Kim, 2023). Furthermore, recognizing the gendered nature of perpetration informs targeted approaches addressing underlying masculinities and social norms that perpetuate sexual violence (Flood, 2020).

The fifth specific objective of this study was to examine sociodemographic correlates—specifically gender, age, and sexual orientation—using a cross-sectional analytical framework. Concerning gender, women consistently reported significantly higher rates of sexual violence: they were more likely than men to experience PSV and SH, including across most individual item analyses, where female participants showed elevated prevalence. In logistic regression models, women had nearly three times greater odds of reporting SH than men. These findings are consistent with global evidence indicating that women bear a disproportionate burden of sexual victimization across settings (Abrahams et al., 2014; Garcia-Moreno et al., 2006; Walters et al., 2013). This heightened vulnerability has been attributed to multiple intersecting factors, including entrenched gender norms, structural inequalities, and power asymmetries within interpersonal relationships. From a psychological standpoint, sociocognitive models suggest that women are often socialized into roles of submission and relational compliance, which may increase their risk of coercion or boundary violations. Furthermore, ecological frameworks of sexual violence emphasize the influence of macrosocial factors—such as gendered socialization and normalization of aggressive male behavior—in perpetuating patterns of victimization. These findings highlight the urgent need for prevention strategies that not only target individual-level skills (e.g., assertiveness, risk detection) but also challenge cultural norms and institutional dynamics that enable sexual violence to persist.

Regarding age-related correlates, the findings of the present study reveal a differentiated developmental pattern of sexual victimization among young adults aged 17 to 30. Although the highest prevalence of PSV was observed in the 20–24 age group, only participants aged 25–30 showed a statistically significant increased likelihood of reporting PSV compared to younger cohorts. Conversely, NPSV and SH were more prevalent among younger cohorts, particularly those aged 17 to 24. This pattern, notably reflected in behaviors such as inappropriate sexual messaging, suggests that distinct forms of sexual violence manifest unevenly across developmental stages. These findings align with Arnett’s (2000) theory of emerging adulthood, which posits that this life stage is characterized by increased relational exploration, exposure to novel social dynamics, and ongoing identity formation—factors that may heighten vulnerability to specific forms of violence. Recent empirical evidence supports these differential patterns. For instance, Boumpa et al. (2024) identified peak incidences of sexual harassment and non-partner sexual violence during late adolescence, whereas intimate partner violence tends to increase in subsequent developmental phases. Similarly, Turanovic (2023) indicated that adolescence and emerging adulthood represent critical periods during which the risk of sexual victimization presents with distinct trajectories and cumulative effects. From a practical standpoint, these results underscore the necessity of tailoring prevention strategies to developmental timing (Baird et al., 2025).

With respect to analysis of sexual orientation, the findings indicate that individuals identifying as LGBTIQ+ demonstrate significantly elevated prevalence rates of NPSV and SH relative to their heterosexual peers, whereas no statistically significant differences were observed in PSV across sexual orientation groups. This pattern corroborates extant empirical evidence pointing to heightened susceptibility to diverse forms of sexual victimization among sexual and gender minority populations (Paquette et al., 2021; Walters et al., 2013). The notably increased odds ratio for SH in LGBTIQ+ participants underscores the endemic and pervasive nature of this form of victimization within marginalized sexual minority communities. These results align conceptually with minority stress theory, which articulates that chronic exposure to social stressors—including stigma, discrimination, and systemic marginalization—potentiates vulnerability to adverse psychosocial outcomes, including increased victimization risk (Dworkin et al., 2018; Meyer, 2003). Furthermore, intersectional and structural models elucidate how compounding systems of oppression intensify susceptibility, especially within sociocultural contexts marked by exclusion and institutional hostility toward sexual and gender minorities (Nacht et al., 2025). Item-level analyses reveal that recurrent sexual violence behaviors—such as persistent or lewd gazes, unwanted physical contact, and offensive sexual comments—disproportionately impact LGBTIQ+ individuals, reflecting complex interplay between individual-level experiences and broader systemic factors. Collectively, these findings emphasize the imperative for the design and implementation of intersectionally informed, trauma-sensitive prevention and intervention frameworks that holistically address the multifaceted risk environment faced by sexual and gender minority populations.

Overall, these findings have significant implications for the development of prevention and response strategies. During late adolescence, it is imperative to implement evidence-based educational programs such as “Safe Dates” and “Shifting Boundaries,” which have demonstrated effectiveness in reducing violence among adolescents (De La Rue et al., 2014). In emerging adulthood, tailored programs designed for university and workplace settings, such as “Dating Matters,” should be promoted (Taylor & Mumford, 2016). Furthermore, the heightened vulnerability of LGBTIQ+ individuals underscores the need to create inclusive and safe spaces, as highlighted by recent studies on sexual violence within this population (Closson et al., 2023).

However, the findings presented should be interpreted with caution, as this study also highlights methodological limitations that warrant consideration. The cross-sectional design restricts the ability to infer causal relationships, while reliance on self-report measures may introduce social desirability bias and lead to underestimation of the true prevalence (Tourangeau & Yan, 2007). Moreover, the absence of assessment of variables such as alcohol consumption, social media use, or prior victimization history may have constrained a comprehensive understanding of the phenomenon. Importantly, the analysis of PSV did not differentiate between participants with and without a history of romantic relationships, which may have influenced prevalence estimates. Future research should also prioritize the role of digital environments and social media in shaping patterns of sexual violence among youth, given their increasing relevance in interpersonal interactions and risk exposure. Moreover, the increasing prevalence of digital sexual violence—manifested through online harassment, non-consensual image sharing, and coercive sexting—warrants dedicated research efforts to understand its dynamics and psychological impact among youth.

Furthermore, greater emphasis is needed on digital sexual violence, particularly salient among young populations (Ybarra et al., 2016), as well as on the long-term psychological consequences, in accordance with the recommendations of Hailes et al. (2019). In any case, this study makes a significant contribution by providing a comprehensive overview of sexual violence among Spanish youth across three age cohorts, encompassing both traditional manifestations and emerging risks. The integration of an ecological and developmental perspective allows not only for the description of the problem’s magnitude but also for the provision of a framework aimed at preventive action. The evidence presented herein reinforces the urgency for informed public policies and underscores the need to sustain ongoing research and prevention efforts in this critical area of public health and human rights.

## 5. Conclusions

“I’d do things to you that you can’t even imagine, but you’d love them …”. This statement encapsulates the complex psychosocial dynamics underlying sexual violence among Spanish youth. Situating #MeToo within this context underscores the urgent need to understand and address the multifaceted nature of victimization, particularly among women and LGBTIQ+ populations, from a comprehensive psychological and social perspective. This study provides updated empirical evidence confirming the high prevalence of sexual violence—both partner and non-partner—as well as sexual harassment within this age group. These findings highlight the critical importance of developing preventive and therapeutic interventions grounded in scientific evidence and tailored to the developmental and psychosocial characteristics of late adolescence and emerging adulthood.

From clinical and educational standpoints, the implementation of evidence-based programs—such as bystander intervention training and curricula emphasizing informed consent—has demonstrated efficacy in reducing sexual violence incidence in university settings. Institutions that engage with youth—including universities, mental health services, and community organizations—must adopt trauma-informed care protocols that ensure safe environments for disclosure and reporting while promoting psychological resilience. At the socio-political level, public policies should be informed by disaggregated and rigorous data to optimize resource allocation and design prevention strategies that consider the diversity of victimization experiences and associated risk factors. Socially inclusive, intersectional approaches—such as tailored programs for LGBTIQ+ youth and culturally competent awareness campaigns—have shown promise in mitigating risk and enhancing access to support services.

Moreover, providing psychological care grounded in empirical models, including trauma-focused cognitive–behavioral therapy and psychoeducational support groups, is essential to facilitate comprehensive recovery, particularly for individuals facing multiple vulnerabilities. In sum, situating #MeToo among Spanish youth demands a commitment to psychological science as a guide for social, clinical, and educational action. Only through systematic, evidence-based interventions can safer and more respectful environments be fostered for future generations.

## Figures and Tables

**Table 1:** Prevalence of sexual victimization by timeframe and timing.

Type of Violence	Timeframe	*n*	%
PSV	Lifetime	131	11.9
	Last 12 months	57	5.2
	Before age 15	7	0.6
	After age 15	97	8.8
	Both periods	26	2.4
NPSV	Lifetime	208	18.9
	Last 12 months	87	7.9
	Before age 15	16	1.5
	After age 15	155	14.1
	Both periods	34	3.1

Note. *n* = 1102.

**Table 2:** PSV: prevalence and sociodemographic correlates.

Typology	Time Frame (%)		Gender (%)		*p*	Sexual Orientation (%)		*p*	Age (%)			*p*
	Lifetime	Last 12 m.	Woman	Man		Heterosexual	LGBTIQ+		17–19	20–24	25–30	
Persistent or lewd gazes	79.6	62.8	74.5	5.1	**<0.001**	61.2	18.4	0.742	30.6	42.9	6.1	0.733
Sexually explicit images	55.1	61.1	51.0	4.1	**0.006**	39.8	15.3	0.348	18.4	31.6	5.1	0.867
Offensive sexual jokes/comments	73.5	61.1	64.3	9.2	**0.044**	56.1	17.3	0.742	28.6	38.8	6.1	0.808
Inappropriate suggestions	57.1	60.7	52.0	5.1	**0.004**	39.8	17.3	0.092	19.4	32.7	5.1	0.746
Unwanted physical contact	73.5	55.6	63.3	10.2	0.162	56.1	17.3	0.742	24.5	43.9	5.1	0.598
Sexual innuendos on social media	54.1	54.7	51.0	3.1	**<0.001**	38.8	15.3	0.257	17.3	31.6	5.1	0.896
Inappropriate sexual messages	50.0	57.1	45.9	4.1	**0.006**	35.7	14.3	0.322	16.3	29.6	4.1	0.720
Threats following sexual rejection	20.4	50.0	19.4	1.0	0.221	16.3	4.1	0.398	8.2	10.2	2.0	0.905
Indecent exposure	35.7	54.3	30.6	5.1	0.738	27.6	8.2	0.990	10.2	21.4	4.1	0.606
Forced to watch pornography	11.2	36.4	10.2	1.0	0.575	7.1	4.1	0.343	2.0	7.1	2.0	0.423

Note. Significant differences are in bold type (*p* < 0.05).

**Table 3:** NPSV: prevalence and sociodemographic correlates.

Typology	Time Frame (%)		Gender (%)		*p*	Sexual Orientation (%)		*p*	Age (%)			*p*
	Lifetime	Last 12 m.	Woman	Man		Heterosexual	LGBTIQ+		17–19	20–24	25–30	
Persistent or lewd gazes	63.1	58.5	55.3	7.8	**<0.001**	51.4	11.7	0.921	26.1	34.9	2.2	0.353
Sexually explicit images	29.3	63.8	24.8	4.5	**<0.001**	21.4	7.9	**<0.001**	11.7	17.1	0.5	0.089
Offensive sexual jokes/comments	46.4	59.1	36.3	10.1	**<0.001**	36.0	10.4	**0.004**	19.7	25.7	1.0	0.215
Inappropriate suggestions	31.6	61.1	26.7	4.9	**<0.001**	23.5	8.1	**<0.001**	12.1	18.4	1.1	0.095
Unwanted physical contact	42.7	62.3	34.1	8.6	**<0.001**	32.9	9.8	**0.003**	19.0	22.5	1.2	0.600
Sexual innuendos on social media	30.2	64.9	25.2	5.0	**<0.001**	22.1	8.1	**<0.001**	12.8	16.5	0.9	0.879
Inappropriate sexual messages	23.6	66.9	18.9	4.7	**<0.001**	17.6	6.0	**0.004**	8.9	14.2	0.5	**0.040**
Threats following sexual rejection	8.6	39.5	5.4	3.2	0.578	6.3	2.3	**0.027**	3.3	5.0	0.3	0.724
Indecent exposure	17.9	54.7	12.6	5.3	0.421	13.8	4.1	0.192	6.7	10.6	0.6	0.113
Forced to watch pornography	5.6	32.1	2.7	2.9	**0.007**	4.3	1.3	0.367	2.5	2.8	0.3	0.649

Note. Significant differences are in bold type (*p* < 0.05).

**Table 4:** Risk of (N)PSV by gender, age, and sexual orientation.

Model	Independent Variable	β	*SE*	*OR*	95% CI		*p*
					LL	UL	
PSV	Gender (woman vs. man)	0.95	0.29	2.58	1.50	4.75	**0.001**
	Age (17–19 vs. 20–24)	0.29	0.23	1.34	0.85	2.13	0.216
	Age (17–19 vs. 25–30)	1.20	0.44	3.33	1.34	7.58	**0.006**
	Age (20–24 vs. 25–30)	−0.91	0.42	0.40	0.18	0.92	**0.031**
	Sexual orientation (LGBTIQ+ vs. heterosexual)	0.12	0.27	1.13	0.65	1.88	0.654
	Intercept	−3.36	0.31	0.04	0.02	0.06	**<0.001**
NPSV	Gender (woman vs. man)	1.80	0.15	6.04	4.56	8.07	**<0.001**
	Age (17–19 vs. 20–24)	0.25	0.14	1.29	0.98	1.69	0.068
	Age (17–19 vs. 25–30)	−0.27	0.35	0.76	0.39	1.51	0.435
	Age (20–24 vs. 25–30)	0.52	0.34	1.69	0.86	3.31	0.129
	Sexual orientation (LGBTIQ+ vs. heterosexual)	0.25	0.18	1.29	0.92	1.82	0.149
	Intercept	−1.10	0.15	0.33	0.25	0.44	**<0.001**

Note. Estimates based on binomial logistic regression models (GLM with binomial family). β = estimate; *SE* = standard error; OR = odd ratio; 95% CI = 95% confidence interval; LL = lower limit; UL = upper limit. Significant differences are in bold type (*p* < 0.05).

**Table 5:** Perpetrator’s gender.

Perpetrator	Man		Woman	
	*n*	%	*n*	%
Parent	14	2	5	3.6
Parent’s partner	7	1	7	5.1
Sibling or step-sibling	7	1	1	0.7
Other relative	16	2.2	3	2.2
Friend	154	21.5	29	21.2
Someone at work	18	2.5	6	4.4
Classmate	72	10.1	14	10.2
Professor	9	1.3	2	1.5
Neighbor	19	2.7	5	3.6
Religiously affiliated individual	3	0.4	5	3.6
Known by sight	218	30.5	26	19
Stranger	178	24.9	34	24.8

**Table 6:** Lifetime prevalence of SH.

Informer		“No”		“Yes”		“Prefer Not to Say”	
		*n*	%	*n*	%	*n*	%
Gender	Man	149	45.7	177	54.3	19	5.8
	Woman	55	8.3	630	77.9	69	6.7
Age	17–19 years	97	20.6	338	71.7	36	7.6
	20–24 years	98	16.8	438	72.5	49	8.4
	25–30 years	9	20.9	31	66.0	3	6.9
Sexual orientation	Heterosexual	616	68.8	270	30.2	9	1.0
	LGTBIQ+	104	51.0	100	49.0	0	-

**Table 7:** SH: prevalence and sociodemographic correlates.

Typology	Time Frame (%)		Gender (%)		*p*	Sexual Orientation (%)		*p*	Age (%)			*p*
	Lifetime	Last 12 m.	Woman	Man		Heterosexual	LGBTIQ+		17–19	20–24	25–30	
Forced to have sex	4.2	69.6	3.7	0.5	**0.009**	2.7	1.5	**0.006**	1.4	2.7	0.1	**0.033**
Sex under influence of substances	9.2	68.3	6.5	2.7	0.519	5.7	3.5	**<0.001**	2.7	6.1	0.4	**0.050**
Unwanted sex due to fear	7.6	68.7	6.6	1.0	**<0.001**	4.5	3.0	**<0.001**	2.6	4.7	0.2	0.168
Forced to have sex against one’s will	8.2	63.3	6.9	1.3	**0.003**	5.4	2.8	**<0.001**	2.4	5.4	0.5	0.063
Attempted to force sex	12.8	73.0	11.5	1.4	**<0.001**	8.8	4.0	**<0.001**	5.1	7.1	0.6	0.485
Touching of intimate body parts	25.1	65.6	21.1	4.0	**<0.001**	17.9	7.2	**<0.001**	8.6	15.7	0.9	**0.006**
Forced to touch intimate body parts	12.0	61.4	10.5	1.5	**<0.001**	8.8	3.2	**0.043**	4.0	7.6	0.4	**<0.001**

Note. Significant differences are in bold type (*p* < 0.05).

**Table 8:** Risk of SH by gender, age and sexual orientation.

Independent Variable	β	*SE*	*OR*	95% CI		*p*
				LL	UL	
Gender (woman vs. man)	1.02	0.16	2.78	2.05	3.81	**<0.001**
Age (17–19 vs. 20–24)	0.50	0.14	1.65	1.26	2.16	**<0.001**
Age (17–19 vs. 25–30)	0.05	0.37	1.05	0.50	2.10	0.895
Age (20–24 vs. 25–30)	0.45	0.36	1.57	0.77	3.18	0.212
Sexual orientation (LGBTIQ+ vs. heterosexual)	0.69	0.16	2.00	1.45	2.75	**<0.001**
Intercept	−1.83	0.17	0.16	0.12	0.22	**<0.001**

Note. Estimates based on binomial logistic regression models (GLM with binomial family). β = estimate; *SE* = standard error; OR = odd ratio; 95% CI = 95% confidence interval; LL = lower limit; UL = upper limit. Significant differences are in bold type (*p* < 0.05).

## Data Availability

The data that support the findings of this study are available from the corresponding author upon reasonable request.

## Abbreviations

NPSV	Non-Partner Sexual Violence
PSV	Partner Sexual Violence
SH	Sexual Harassment
